# Genomic and Proteomic Study of the Inflammatory Pathway in Patients With Atrial Fibrillation and Cardiometabolic Syndrome

**DOI:** 10.3389/fcvm.2020.613271

**Published:** 2020-12-02

**Authors:** Hana A. Itani, Miran A. Jaffa, Joseph Elias, Mohammad Sabra, Patrick Zakka, Jad Ballout, Amira Bekdash, Rand Ibrahim, Moustafa Al Hariri, Mirna Ghemrawi, Bernard Abi-Saleh, Maurice Khoury, Samir Alam, Rami Mahfouz, Ayad A. Jaffa, Sami T. Azar, Marwan M. Refaat

**Affiliations:** ^1^Faculty of Medicine, American University of Beirut, Beirut, Lebanon; ^2^Department of Medicine, Clinical pharmacology, Vanderbilt University Medical Center, Nashville, TN, United States; ^3^Epidemiology and Population Health Department, Faculty of Health Sciences, American University of Beirut, Beirut, Lebanon; ^4^Department of Internal Medicine, Cardiology Division, American University of Beirut Faculty of Medicine and Medical Center, Beirut, Lebanon; ^5^Department of Internal Medicine, Emory University Hospital, Atlanta, GA, United States; ^6^Department of Emergency Medicine, American University of Beirut Faculty of Medicine and Medical Center, Beirut, Lebanon; ^7^Department of Pathology and Laboratory Medicine, American University of Beirut Faculty of Medicine and Medical Center, Beirut, Lebanon; ^8^Department of Biochemistry and Molecular Genetics, American University of Beirut Faculty of Medicine, Beirut, Lebanon; ^9^Department of Internal Medicine, Endocrinology Diabetes and Metabolism Division, American University of Beirut Faculty of Medicine and Medical Center, Beirut, Lebanon

**Keywords:** cardiac arrhythmia, cardiovascular diseases, heart diseases, atrial fibrillation, inflammatory markers, metabolic syndrome, gene expression

## Abstract

Atrial fibrillation (AF) and cardiometabolic syndrome (CMS) have been linked to inflammation and fibrosis. However, it is still unknown which inflammatory cytokines contribute to the pathogenesis of AF. Furthermore, cardiometabolic syndrome (CMS) risk factors such as obesity, hypertension, insulin resistance/glucose intolerance are also associated with inflammation and increased level of cytokines and adipokines. We hypothesized that the inflammatory immune response is exacerbated in patients with both AF and CMS compared to either AF or CMS alone. We investigated inflammatory cytokines and fibrotic markers as well as cytokine genetic profiles in patients with lone AF and CMS. CMS, lone AF patients, patients with both lone AF and CMS, and control patients were recruited. Genetic polymorphisms in inflammatory and fibrotic markers were assessed. Serum levels of connective tissue growth factor (CTGF) were tested along with other inflammatory markers including platelet-to-lymphocyte ratio (PLR), monocyte-to-HDL ratio (MHR) in three groups of AF+CMS, AF, and CMS patients. There was a trend in the CTGF levels for statistical significance between the AF and AF+CMS group (*P* = 0.084). Genotyping showed high percentages of patients in all groups with high secretor genotypes of Interleukin-6 (IL-6) (*P* = 0.037). Genotyping of IFN-γ and IL-10 at high level showed an increase in expression in the AF + CMS group compared to AF and CMS alone suggesting an imbalance between the inflammatory and anti-inflammatory cytokines which is exacerbated by AF. Serum cytokine inflammatory cytokine levels showed that IL-4, IL-5, IL-10, IL-17F, and IL-22 were significant between the AF, AF+CMS, and CMS patients. Combination of both CMS and AF may be associated with a higher degree of inflammation than what is seen in either CMS or AF alone. Thus, the identification of a biomarker capable of identifying metabolic syndrome associated with disease will help in identification of a therapeutic target in treating this devastating disease.

## Introduction

Cardiometabolic syndrome (CMS) is a substantial cause of worldwide morbidity and mortality and represents a cluster of metabolic abnormalities and significant cardiovascular disease risk factors that can lead to non-communicable diseases (NCD) (1, 2). It has been recently reported that 25% of adults in the US have metabolic syndrome which is attributed to a higher risk for developing atherosclerotic CVD and premature cardiovascular mortality (2–4). Metabolic disorders in metabolic syndrome are associated with insulin resistance, visceral adiposity, obesity, dyslipidemia, endothelial dysfunction, and hypertension with prominent end-organ damages in the cardiovascular system, pancreas, and liver (4, 5). Mitochondrial dysfunction, chronic inflammation, gut microbiome, genetic variation, and environmental contaminants are factors that contribute to the pathogenesis of metabolic syndrome and its transition to CVD (2, 4). Genome-wide association studies (GWAS) studies performed on metabolic syndrome identified genetic variants that are involved in glucose and lipid metabolism (6). Recent studies revealed the importance of the pro-inflammatory state in metabolic syndrome through mediating vascular dysfunction (5). In addition, high levels of serum TNF-α and IL-6 have shown to be linked to obesity and insulin resistance which are key players in metabolic syndrome (2). Thus, far, there is no single biomarker capable of identifying metabolic syndrome, yet a promising panel of biomarkers were shown to be associated with disease (7). Elevated levels of pro-inflammatory cytokines (IL-6, TNF-α), adipokines (Leptin, adiponectin, ghrelin), LAR, prothrombotic factors (PAI-1), uric acid, and pro-oxidant (oxidized LDL) coupled with lower levels of anti-inflammatory cytokines (IL-10) and antioxidant factors (PON-1) are noted in metabolic syndrome (7).

Atrial fibrillation (AF), on the other hand, is a major arrhythmia defined as fast and disorganized electrical excitation of the atria affecting cardiac function (8, 9). AF is considered as a risk factor for stroke, as more than 15% of strokes in the US are due to AF (8). AF together with hypertension could lead to thromboembolic complications and congestive heart failure (10). As a consequence, AF is correlated with increased morbidity and mortality (8). Current treatments of AF target stroke prevention, rate and rhythm control (8, 9). Many research studies proposed gene therapy as a method to reduce the expression of abnormal genes related to the pathogenesis of AF such as ion channels, gap junctions, parasympathetic nervous system, and fibrosis (11). In addition, the impact of gender differences on AF is found to be prominent. Significant differences between women and men with AF are related to AF mechanisms, therapy response stroke risk reduction strategies, as well as other outcomes such as quality of life (8).

Type II diabetes, a major component of CMS, is considered a strong risk factor for AF, and metabolic syndrome has been associated with AF recently (12). Most metabolic syndrome components are found to have an additive effect on the risk of AF (13, 14). Oxidative stress and inflammation are common components related to the pathogenesis of both metabolic syndrome and AF (13). Other components such as hypertension, dyslipidemia, and abdominal adiposity increase the risk of new-onset AF (14, 15). Given the circumstances, studies determined the strong association of low HDL cholesterol, elevated fasting glucose levels in increasing the risk of AF (14, 15). Furthermore, elevated levels of inflammatory mediators were encountered in atrial biopsies from patients with AF (16). The synergistic effect of CMS components is capable of exacerbating the outcome of AF and coronary heart diseases leading to cardiovascular mortality (17). Regardless, the exact mechanism behind the association between CMS and AF is still insufficient and further studies are required to understand the etiology and relation between CMS in AF (15). Increasing evidence supports the role of inflammation-associated cytokines and chemokines in the pathogenesis of atrial fibrillation (AF). Several pharmacological interventions with established anti-inflammatory effects and corticosteroids are associated with AF. Many inflammatory pathways lead to electrical and structural remodeling of the atria that predispose to AF. The Infiltration of immune cells including macrophages and T lymphocytes releasing inflammatory cytokines and inflammatory mediators such as C-reactive protein (CRP) enhance the inflammatory response in cardiac tissue are major players in the development of AF. In the current study, we investigated the inflammatory profile of fibrotic markers as well as cytokine genetic profiles in patients with lone AF, CMS, and combination of both AF and CMS. We further compared the plasma levels of inflammatory cytokines in those patients. The understanding of the inflammatory pathophysiology in AF and CMS patients will help us to identify specific and potential therapeutic strategies for the prevention of AF.

## Materials and Methods

### Study Population

This prospective study included four study groups: patients with CMS and lone AF, patients with CMS without lone AF, patients with lone AF without CMS, and a control group with neither CMS nor lone AF (previously healthy). According to the modified NCEP ATP III criteria, the following are cardiometabolic risk factors and the presence of any three of these factors is required for a diagnosis of CMS: Abdominal Obesity [>40”/102 cm in men and >35”/88 cm in women], hypertriglyceridemia (≥150 mg/dL or ≥1.7 mmol/L), reduced HDL-C [<40 mg/dL (1.03 mmol/L) in men and <50 mg/dL (1.29 mmol/L) in women], elevated blood pressure [≥130/85 mm Hg or use of medication for hypertension], impaired Fasting Glucose [≥100 mg/dL (5.6 mmol/L) or use of medication for hyperglycemia]. Subjects were excluded from this study if they have a history of moderate or severe mitral regurgitation or stenosis, severe aortic stenosis, hyperthyroidism, acute infection, chronic inflammatory diseases, active malignancy, chronic liver disease, cardiomyopathy, or heart failure (EF < 40%) or history of cardiac surgery including CABG or valve replacements.

CMS patients (*n* = 11), lone AF patients (*n* = 27), and patients with both lone AF with CMS (*n* = 33) and Controls (*n* = 4) were recruited for this study from the American University of Beirut Medical Center (AUBMC). All patients provided an informed consent form approved by the Institutional Review Board (IRB) at the AUBMC. Patients were asked to complete a standardized questionnaires to collect baseline information.

### Assessing the Cytokine Genotyping and Expression Levels

The cytokine genotyping is based on PCR-SSP methodology which provides sequence-specific oligonucleotide primers for amplification of selected TNF-α, TGF-β1, IFN-γ, IL-6, and IL-10 alleles and the human β-globin gene by the polymerase chain reaction (PCR). These alleles are known to be associated with the expression level of these factors. The primer pairs are designed to have perfect matches only with a single allele or group of alleles. Under strictly controlled PCR conditions, perfectly matched primer pairs result in the amplification of target sequences (i.e., a positive result), while mismatched primer pairs do not result in amplification (i.e., a negative result).

Pre-optimized primers are presented (dried) in different wells of a 96-well 0.2 ml thin-walled tube tray for PCR and are ready for the addition of DNA samples, recombinant Taq polymerase, and specially formulated dNTP-buffer mix (D-mix). Each tray includes a negative control reaction tube that detects the presence of the internal control product generated by the tray. After the PCR process, the amplified DNA fragments are separated by agarose gel electrophoresis and are visualized by staining with ethidium bromide and exposure to ultraviolet light. Interpretation of PCR-SSP results is based on the presence or absence of a specific amplified DNA fragment. Since amplification during the PCR reaction may be adversely affected by various factors (pipetting errors, poor DNA quality, presence of inhibitors, etc.) an internal control primer pair is included in every PCR reaction. The control primer pair amplifies a conserved region of the human β-globin gene, which is present in all DNA samples and is used to verify the integrity of the PCR reaction. In the presence of a positive typing band (specific amplification of a cytokine allele), the product of the internal control primer may be weak or absent due to the difference in concentration and melting temperatures between the specific primer pairs and the internal control primer pair. The amplified DNA fragments of the specific cytokine primer pairs are smaller than the product of the internal control primer pair, but larger than the diffuse, unincorporated primer band. Thus, a positive reaction for a specific cytokine allele or allele group is visualized on the gel as an amplified DNA fragment between the internal control product band and the unincorporated primer band.

### Measurement of Human Cytokine Levels and Fibrotic Markers

Serum samples were tested for 13 human inflammatory cytokines using the LEGENDplex^TM^ Human Th cytokine panel for interleukins (ILs, pg/mL) IL-2, 4, 5, 6, 9, 10, 13, 17A, 17F, 21, 22, IFN-γ and TNF-α, which are collectively secreted by Th1, Th2, Th9, Th17, Th22, and T follicular cells (Biolegend). Samples were treated following the manufacturer's instructions and measured with a BD FACS Aria™SORP cell sorter (BD Biosciences). Analysis was done using Data Analysis V8.0 software. Serum levels of IL-18, connective tissue growth factor (CTGF) were tested along with other inflammatory markers including platelet-to-lymphocyte ratio (PLR) and monocyte-to-HDL ratio (MHR).

### Statistical Analysis

Our analysis was initiated by carrying out descriptive analysis to provide a summary statistics to all parameters. Continuous parameters were summarized using mean and standard deviation, and categorical parameters were presented using count and percent. To assess the crude unadjusted associations between the IL levels and the groups (AF, AF+CMS, and CMS), we employed the conservative non-parametric tests Mann-Whitney *U* test for the comparisons between the two groups, and Kruskal Wallis for comparisons between multiple groups. Our adjusted analysis was carried out to model the ILs as a function of the groups (AF, AF+CMS, and CMS), and selected clinical and demographic characteristics. In this respect, multiple linear regression was carried out using the logarithmic scale of the ILs employed to achieve symmetry in the outcomes. The AF group was chosen as the reference group. Significance level was chosen to be 0.05, and ILs found to be significantly associated with the groups (AF, AF+CMS, and CMS) at the level of unadjusted analysis were further considered in the multivariable linear regression. To assess the association between categorical variables as in the groups (AF, AF+CMS, CMS) and the stratified genetic polymorphisms (High, Intermediate, Low), we employed the Fisher's Exact test to be on the conservative side when reporting the p-values, given that some of the expected cells were <5 (**Table 2**). In **Table 3**, the mean of ILs are presented across the different groups along with the corresponding P-values obtained using the Kruskal-Wallis test, and in **Table 4** results of the multivariable adjusted analysis for the clinical and demographic characteristics are displayed In Appendix 1-Table a, a detailed summary statistics of the ILs are presented across the different groups. In Appendix 1-Table b, the P-values corresponding to the Mann-Whitney *U* tests unadjusted and adjusted for multiple comparisons are presented between the groups as well as the P-values for the Kruskal Wallis tests for multiple group comparisons. Box-plots pertaining to the ILs that were significantly associated with the groups (AF, AF+CMS, and CMS) were included in Appendix 2 – Figure 1(a–e). Our data analysis was conducted using SPSS 23, and STATA 14.

## Results

### Characteristics of the Study Population

Table 1 shows the baseline characteristics or parameters for the study population (AF, AF+CMS, CMS) and are described in terms of mean for continuous data, and percentages for categorical data. These parameters included HDL-C, LDL-C, total cholesterol, systolic blood pressure (SBP), diastolic Blood pressure (DBP), age, gender and smoking status. At baseline, the study population included relatively equal distribution of women (58.3, 54.8, and 36.36%) and men (41.67, 45.16, and 63.64%) among the AF, AF+CMS, and CMS groups.

**Table 1:** Baseline characteristics of the study population described in terms of mean (standard deviation) for continuous data, and percentages for categorical data.

**Characteristic**	**AF**	**CMS**	**AF + CMS**
Age			
Male	58.21 (22.66)	67.25 (7.97)	69.65 (12.65)
Female	67.5 (16.87)	57.57 (12.27)	76.43 (7.21)
All	62.08 (20.58)	61.09 (11.55)	72.71 (10.49)
Gender			
Male	58.33%	36.36%	54.84%
Female	41.67%	63.64%	45.16%
BMI			
Normal weight = 18.5–24.9	38.89%	11.11%	10.71%
Overweight = 25–29.9	33.33%	33.33%	39.29%
Obesity = BMI of 30 or greater	27.78%	55.56%	50%
Average	27.11 (5.75)	31.19 (4.7)	30.61 (6.26)
Blood pressure (mmHg)			
SBP	128.35 (12.92)	143.40 (13.37)	127.52 (19.34)
DBP	77.04 (15.51)	77.70 (12.14)	69.29 (11.56)
Mean	94 (11.75)	99.6 (8.31)	88.74 (13.28)
HDL	56.17 (16.23)	41.1 (11.91)	42.97 (13.17)
LDL	110.07 (31.05)	87.9 (29.99)	92.5 (33.03)
Total cholesterol	180.83 (37.25)	174.8 (51.48)	157.57 (36.95)
Smoking			
Yes	17.39%	20.00%	16.13%
Former	21.74%	0%	9.68%
No	60.87%	80.00%	74.19%
Platelet-lymphocyte ratio (PLR)	135.66 (66.37)	145.06 (63.62)	127.63 (53.92)
Monocyte-HDL ratio (MHR)	9.78 (4.94)	16.71 (10.8)	17.63 (12.4)
CTGF level (pg/ml)	2598.25 (1918.02)	2314.07 (1781.41)	1638.48 (1634.13)

### Gene Expression and Fibrotic Markers Tested in Lone AF, CMS, and AF + CMS Patients

To assess genetic polymorphisms in inflammatory markers such as tumor necrosis factor alpha (TNF-α), transforming growth factor beta 1 (TGF-β1), interleukin 10 (IL-10), interleukin 6 (IL-6), interferon gamma (IFN-γ), DNA extraction was performed from lone AF, CMS, AF + CMS, and control patients whose clinical data is described in Table 1. The levels of these genetic polymorphisms were stratified to high, intermediate and low (Table 2) and the associations between these classes and the groups (AF, AF+CMS, and CMS) were determined using Fisher's Exact tests to be on the conservative side when reporting significant associations especially in the presence of small cell count and expected cells that are <5. Genotyping showed high percentages of patients in the AF, AF+CMS, CMS groups with high secretor genotypes of TGF-β1 (81.48, 70, 72.73%, Fisher's exact test *P* = 0.19) but it did not reach statistical significance. Genotyping showed high percentages of patients in the AF, AF+CMS, CMS groups with high secretor genotypes of IL-6 (95.65, 60, 86.67%, Fisher's exact test *P* = 0.037), respectively as shown in Table 2. IL-10 stratified levels were associated with the AF, AF+CMS, CMS groups with Fisher's exact test *P* = 0.05 (Table 2). Genotyping of IFN-γ and IL-10 at high level showed an increase in expression in the AF + CMS group compared to AF and CMS alone suggesting an imbalance between the inflammatory and anti-inflammatory cytokines which is exacerbated by AF.

**Table 2:** Genetic polymorphisms in inflammatory (TNFα, TGF-β, IL-10, IL-6, and IFN-γ) stratified to high, intermediate and low levels in patients with AF, CMS, and AF +CMS.

**Gene expression**	**Marker**	**Level**	**AF%**	**CMS%**	**AF + CMS%**	***P*^¥^**
	Tumor necrosis factor alpha (TNF-α)	High	18.52	10	15.15	0.999
		Intermediate	0	0	3.03	
		Low	81.48	90	81.82	
	Transforming growth factor beta 1 (TGF-β1)	High	81.48	70	72.73	0.19
		Intermediate	18.52	10	24.24	
		Low	0	20	3.03	
	Interleukin 10 (IL-10)	High	7.41	0	18.18	0.05^*^
		Intermediate	51.85	20	54.55	
		Low	40.74	80	27.27	
	Interleukin 6 (IL-6)	High	95.65	60	86.67	0.037^*^
		Intermediate	4.35	40	13.33	
	Interferon gamma (IFN-γ)	High	22.22	30	33.33	0.226
		Intermediate	55.56	20	48.48	
		Low	22.22	50	18.18	

¥ *P-value from Fisher's Exact test*.

* *Statistical significance with P < = 0.05 was reached*.

### Evidence for Inflammation in Atrial Fibrillation and Cardiometabolic Syndrome

Serum levels of connective tissue growth factor were tested along with other inflammatory fibrotic markers including connective tissue growth factor (CTGF), platelet-to-lymphocyte ratio (PLR) and monocyte-to-HDL ratio (MHR). CTGF levels were not statistically significant among the groups (Kruskal-Wallis test *P* = 0.227); there was a trend in the CTGF levels between the AF and AF+CMS group (Mann-Whitney test *P* = 0.084). PLR was highest in groups with CMS and lowest in the AF group but was not statistically significant (Kruskal-Wallis test *P* = 0.75, and Mann-Whitney test *P* > 0.05) for all group comparisons. The monocyte-to-HDL ratio (MHR) was significantly different between groups (Krukal-Wallis test *P* = 0.005). MHR was significantly different in particular between AF and AF+CMS groups (unadjusted Mann-Whitney test *P* = 0.002 and adjusted *P* = 0.006 for multiple comparisons). Significant difference in MHR was also detected between AF and CMS groups with Mann-Whitney test *P* = 0.029 unadjusted for multiple comparisons; however, the Mann-Whitney test adjusted for multiple comparisons was not significant with *P* = 0.087. No significant difference in MHR was detected between the AF+CMS and the CMS group (unadjusted Mann-Whitney test, *P* = 0.816).

**Figure 1:**
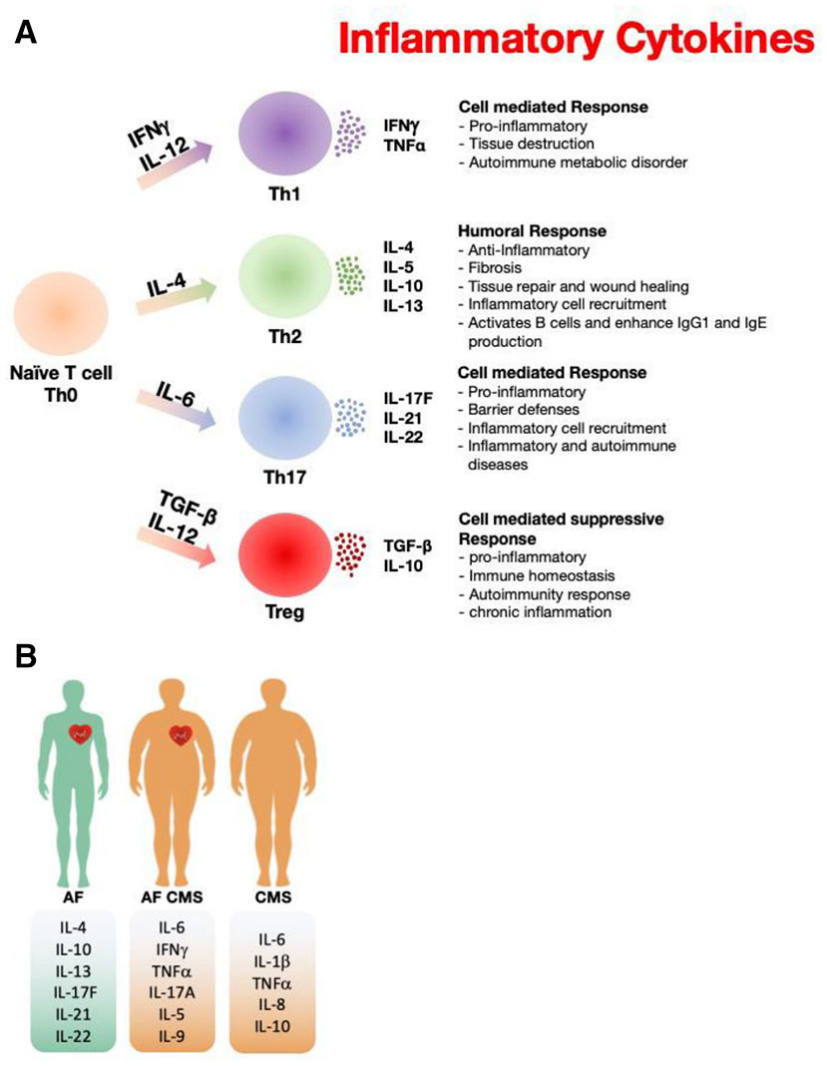
Paradigm illustrating the role of inflammatory markers in atrial fibrillation and cardiometabolic syndrome. **(A)** The inflammatory cytokine released from different types of T cells that have been reported in AF and CMS. **(B)** Differences of the inflammatory cytokine profile among AF, CMS, and AF+CMS recruited patients.

To assess the level of inflammatory cytokines in the above cohort, human serum cytokine levels were quantified in patients with lone AF, CMS, and AF + CMS. Serum interleukin levels of IL-4, IL-5, IL-10, and IL-17F and IL-22 were significantly different in all groups (Table 3 and Appendix 1-Tables a,b, Appendix 2 Figures 1a–e). The remaining ILs did not exhibit a significant difference across the groups.

**Table 3:** Inflammatory cytokine mean levels in AF, CMS, and AF + CMS patients along with corresponding standard deviations (SD).

Cytokines^*^	**AF**	**AF + CMS**	**CMS**	***P***
	**Mean (SD)**	**Mean (SD)**	**Mean (SD)**	
IL-17A, pg/mL	0.579 (0.824)	0.785 (1.466)	0.089 (0.076)	0.272
IL-17F, pg/mL	2.052 (1.935)	1.370 (1.802)	0.332 (0.196)	0.005^*^
IFN-γ, pg/mL	4.032 (3.073)	6.392 (7.786)	3.152 (0.962)	0.841
TNF-α, pg/mL	3.074 (1.880)	4.813 (3.594)	3.405 (2.634)	0.250
IL-9, pg/mL	2.426 (1.910)	3.166 (3.017)	1.574 (0.848)	0.273
IL-6, pg/mL	5.166 (2.751)	8.656 (9.107)	4.889 (3.764)	0.566
IL-2, pg/mL	0.463 (0.544)	0.404 (0.587)	0.385 (0.584)	0.750
IL-4, pg/mL	4.290 (2.884)	2.100 (1.859)	0.874 (0.465)	0.000^*^
IL-5, pg/mL	4.497 (2.509)	4.750 (4.098)	2.033 (0.968)	0.037^*^
IL-10, pg/mL	2.167 (0.815)	1.833 (1.117)	1.166 (0.150)	0.003^*^
IL-13, pg/mL	14.367 (13.899)	4.718 (4.438)	-	0.095
IL-21, pg/mL	12.616	9.165	5.304	0.228
IL-22, pg/mL	4.028	3.662	1.078	0.084

* *TNF-α, Tumor Necrosis Factor alpha; IFN-γ, interferon-γ; IL-17A, interleukin 17A; IL-17F, interleukin 17F; IL-21, interleukin 21; IL-22, interleukin 22; IL-10, interleukin 10; IL-9, interleukin 9; IL-6, interleukin 6; IL-4, interleukin 4; Il-13, interleukin 13; IL-2, interleukin 2. Kruskal Wallis P-value*.

* *Statistical significance was reached, P < 0.05*.

### IL-4:

The summary statistics for IL-4 across the three groups: AF, AF+CMS, and CMS depicted in Appendix1-Table A, showed that IL-4 was highest in AF group compared to AF+CMS and CMS group (4.290±2. vs. 2.100±1.859 and 0.874± 0.465 pg/mL, respectively). Kruskal Wallis test was carried out in order to determine if there was an overall group effect on IL-4 levels between the three different groups (AF, AF+CMS, CMS). Two of the groups had significant difference in IL-4 (*P* < 0.0001, Kruskal Wallis; Table 3 and Appendix 1-Table b). To compare the level of IL-4 between all the groups in a pairwise manner (Appendix 1-Table b), we performed Mann-Whitney *U* tests and presented unadjusted and adjusted P-values for multiple comparisons. Our results indicated that there were unadjusted significance in two pairwise comparisons (AF and AF+CMS; AF and CMS (*P* < 0.05), and one insignificant pairwise comparison between AF+CMS and CMS (*P* > 0.05). This indicated that there was a difference in IL-4 between AF and the remaining two groups which are AF+CMS and CMS. IL-4 was higher in AF compared to that of AF+CMS group (4.290 vs. 2.100, unadjusted *P* = 0.001; adjusted *P* = 0.006) as shown in Appendix 1 Tables a,b. In addition, IL-4 was higher in AF compared to the CMS group (4.290 vs. 0.874 pg/mL, unadjusted *P* = 0.0001; adjusted *P* = 0.0001). The remaining comparison, between AF+CMS and CMS were insignificant with Mann-Whitney test unadjusted *P* = 0.06 and adjusted *P* = 0.256, respectively.

### IL-5:

The summary statistics for IL-5 across the three groups: AF, AF+CMS, and CMS displayed in Appendix1-Table a showed that IL_5 was highest in AF+CMS group compared to lone AF and CMS group (4.497±2.509 vs. 4.750±4.098 and 2.033± 0.968 pg/mL, respectively). To determine if there was an overall group effect on IL-5 between the three different groups (AF, AF+CMS, CMS), we performed a Kruskal Wallis test which indicated that at least two groups had significant difference in IL-5 (*P* = 0.037, Table 3 and Appendix 1-Table b). To compare the IL-5 between all the groups in pairwise manner (Appendix 1-Table b), we performed Mann-Whitney *U* tests and presented unadjusted and adjusted P-values for multiple comparisons. Our results showed unadjusted Significance (*P* < 0.05) in one pairwise comparison among AF and CMS groups, and two insignificant in two pairwise comparisons between AF and AF+CMS and between AF+CMS and CMS group (*P* > 0.05). This indicated that IL-5 was higher in AF compared to CMS group (4.497 vs. 2.033 pg/mL, unadjusted *P* = 0.004). However, after we adjusted for the multiple comparisons, AF and CMS groups continued to show significant difference in IL-5 values with Adjusted *P* = 0.034.

### IL-10:

The summary statistics for IL-10 across the three groups AF, AF+CMS, and CMS were depicted in Appendix 1-Table a showed that IL-10 was highest in AF group compared to AF+CMS and the CMS group(2.167±0.815 vs. 1.833±1.117 and 1.166± 0.150 pg/mL, respectively). To determine if there was an overall group effect on IL-10, Kruskal Wallis test (Table 3 and Appendix 1-Table b) was performed that indicating that at least two groups had significant difference in IL-10 (*P* = 0.003). Mann- Whitney *U* test was carried out to compare the IL-10 between all the groups (Appendix 1-Table b) and unadjusted and adjusted P for multiple comparisons were reported. Our results indicate significance in all three pairwise comparisons (unadjusted *P* < 0.05). This indicates that IL-10 was highest in the AF groups compared to the AF+CMS group (2.167 vs. 1.833, unadjusted *P* = 0.035) and highest in AF compared to the CMS group (2.167 vs. 1.166 pg/mL, unadjusted *P* = 0.002). In addition, our results indicated that IL-10 was highest in AF+CMS compared to the CMS group (1.833 vs. 1.166 pg/mL, unadjusted *P* = 0.039). However, after we adjusted for the multiple comparison, only AF and CMS groups had significant difference in the IL-10 (adjusted *P* = 0.003). The remaining comparisons between AF and AF+CMS, and AF+CMS and CMS lost the significance difference that was caught before the adjustment for multiple comparisons. These groups exhibited non-significant difference in the IL-10 after adjustment for multiple comparisons (AF and AF+CMS; AF+CMS and CMS, adjusted *P* = 0.16) (Appendix 1-Table b).

### IL-17F:

The summary statistics for IL-17F across the three groups AF, AF+CMS, and CMS (Appendix 1-Table a) showed that IL_17F was highest in AF group compared to AF+CMS and the CMS group (2.052±1.935 vs. 1.370±1.802 and 0.332± 0.196 pg/mL, respectively). To determine if there was an overall group effect on *IL-17F*, Kruskal Wallis test (Table 3 and Appendix 1-Table b) was carried out between the three different groups AF, AF+CMS, and CMS indicating that at least two groups had significant difference in IL-17F (*P* = 0.005). Mann- Whitney U test was carried out to compare the IL-17F values between all the groups (Appendix 1-Table b), and unadjusted and adjusted P for multiple comparisons were presented. Our results indicate significance in two pairwise comparisons (AF and AF+CMS; AF and CMS, Unadjusted *P* < 0.05) and insignificant pairwise comparison (AF+CMS and CMS, unadjusted *P* = 0.05). This indicated that IL-17F was higher in AF compared to the AF+CMS group (2.052 vs. 1.370 pg/mL, unadjusted *P* = 0.039); and higher in AF compared to the CMS group (2.052 vs. 0.332, unadjusted *P* = 0.001). However, after we adjusted for multiple comparisons, only AF and CMS groups had significant difference in the IL-17F (adjusted *P* = 0.005). The remaining comparisons between AF and AF+CMS groups, and the CMS and AF+CMS groups lost the significant differences that were caught before the adjustment for multiple comparisons and showed non-significant difference in the IL-17F after adjustment for multiple comparisons (multiple comparison, adjusted *P* = 0.102 for the comparisons between AF and AF+CMS, and *P* = 0.326 for the comparisons between AF+CMS and CMS).

### IL-18

IL-18 level was assessed by ELISA and was not significant across the groups AF (262 pg/mL), AF+CMS (234 pg/mL), CMS (191 pg/mL) and control (224 pg/mL).

### IL-22:

The summary statistics for IL-22 across the three groups AF, AF+CMS, and CMS displayed in Appendix 1-Table a, showed that IL_22 was highest in AF group compared to AF+CMS and the CMS group (4.028±4.508 vs. 3.662±4.639 and 1.078± 1.475 pg/mL, respectively). Kruskal Wallis test carried out in order to determine if there was an overall group effect on the IL-22 between the three different groups (Table 3 and Appendix 1-Table b). Our results indicated that none of the groups had significant difference in IL-22 after adjusting for multiple comparisons (*P* = 0.084). IL-22 levels were insignificant between all the groups in pairwise manner (Appendix 1-Table b) *P* > 0.05 in two comparisons (AF and AF+CMS, unadjusted *P* = 0.992 for multiple comparisons; AF and CMS, *P* = 0.076). However, IL-22 was significantly higher in the AF+CMS compared to the CMS group (3.662 vs. 1.078 pg/mL, unadjusted *P* = 0.021 for multiple comparisons). However, this significant difference in IL-22 between the AF+CMS and CMS group was no longer present after adjusting for multiple comparisons.

In line with gene expression data, the cytokine serum levels revealed a trend of high levels of IL-6, IFNγ, TNFα, and IL-17A in blood samples drawn from patients with AF CMS compared to patients with CMS or AF alone (Table 3). These findings suggest that CMS promotes further the inflammatory process in AF patients. To assess the imbalance of inflammation in those patients, we measured cytokines contributing to the anti-inflammatory responses and protective pathways involved in tissue repair. Furthermore, we found that IL-4, IL-10, IL-13, IL-17F were significantly higher in patients with AF compared to AF CMS patients (Table 3). There was a trend in higher levels of IL-21, IL-22 in AF patients alone but didn't reach significance as shown in Table 3. In summary our results showed that when a group effect was present on any IL, this effect was mainly triggered by AF since it was the group that exhibited the difference in ILs when compared to AF+CMS and the CMS groups.

### Multivariable Adjusted Associations Between Cytokine Levels and Patient Clinical Data

To determine whether serum levels of these cytokines correlated with atrial remodeling, we studied correlations between cytokine levels and parameters in the selected cohort of patients. The inflammatory markers that were significantly associated with the groups (AF, AF+CMS, and CMS) were considered in our multivariable analyses that were adjusted for clinical and demographic characteristics. These parameters included HDL, LDL, total cholesterol, SBP, DBP, age, gender and ever smoked. AF was taken as our reference group. The IL were all transformed to logarithmic scale to achieve symmetry and multivariable linear regressions were carried out whereby the ILs were modelled as functions of the aforementioned clinical and demographic patients' characteristics. Significant ILs at the univariable level of analysis which were considered in the multivariable analyses included IL-4, IL-5, IL-10, and IL-17-F. Results of the multivariable analysis were displayed in Table 4.

**Table 4:** Multivariable analysis with IL being the outcome of interest and group (AF, AF +CMS, and CMS) as the main predictor, and clinical and demographic characteristics as covariates to factor for in the analysis.

**IL**	**Predictor**	**Coefficient estimate**	**Standard Error**	***P***	**95% CI for the coefficient estimate**
**IL4 (LOGARITHMIC SCALE)**					
	AF (Ref)	—	—	—	—
	AF+CMS	−0.788	0.315	0.022^*^	−1.449; −0128
	CMS	−2.305	0.553	0.001^*^	−3.464; −1.147
	Gender	−0.427	0.385	0.281	−1.233; 0.379
	Age	0.001	0.107	0.995	−0.0223; 0.022
	Ever smoked	0.268	0.355	0.459	−0.475; 1.012
	HDL	0.021	0.014	0.149	−0.008; 0.051
	LDL	0.006	0.008	0.477	−0.011; 0.022
	Total cholesterol	−0.006	0.007	0.407	−0.021; 0.009
	SBP	0.028	0.015	0.077	−0.003; 0.061
	DBP	−0.019	0.012	0.156	−0.046; 0.007
**IL5 (LOGARITHMIC SCALE)**					
	AF (Ref)	—	—	—	—
	AF+CMS	−0.047	0.259	0.856	−0.591; 0.496
	CMS	−1.153	0.494	0.031^*^	−2.189; −0.118
	Gender	−0.690	0.319	0.044^*^	−1.358; −0.021
	Age	0.004	0.008	0.610	−0.013; 0.022
	Ever smoked	−0.015	0.292	0.958	−0.628; 0.597
	HDL	0.029	0.011	0.021^*^	0.004; 0.053
	LDL	0.007	0.006	0.236	−0.005; 0.021
	Total cholesterol	−0.007	0.006	0.254	−0.019; 0.005
	SBP	0.021	0.012	0.107	−0.005; 0.047
	DBP	−0.012	0.011	0.263	−0.034; 0.010
**IL10 (LOGARITHMIC SCALE)**					
	AF (Ref)	–	-	-	-
	AF+CMS	−0.127	0.133	0.345	−0.396; 0.141
	CMS	−0.374	0.197	0.064	−0.772; 0.023
	Gender	−0.038	0.138	0.783	−0.317; 0.240
	Age	−0.008	0.004	0.059	−0.016; 0.000
	Ever smoked	−0.212	0.126	0.100	−0.467; 0.041
	HDL	0.011	0.005	0.038^*^	0.001; 0.021
	LDL	0.003	0.003	0.321	−0.003; 0.009
	Total cholesterol	−0.002	0.003	0.361	−0.008; 0.003
	SBP	−0.001	0.004	0.865	−0.009; 0.008
	DBP	−0.004	0.005	0.382	−0.015; 0.006
**IL17_F (LOGARITHMIC SCALE)**					
	AF (Ref)	-	-	-	-
	AF+CMS	−0.409	0.432	0.355	−1.312; 0.492
	CMS	−1.795	0.751	0.027^*^	−3.363; −0.227
	Gender	−0.959	0.529	0.085	−2.063; 0.143
	Age	−0.002	0.014	0.864	−0.032; 0.027
	Ever smoked	0.132	0.487	0.789	−0.885; 1.149
	HDL	0.064	0.019	0.003^*^	0.023; 0.104
	LDL	0.021	0.010	0.048^*^	0.001; 0.042
	Total cholesterol	−0.017	0.009	0.097	−0.037; 0.003
	SBP	0.044	0.020	0.047^*^	0.001; 0.088
	DBP	−0.016	0.017	0.375	−0.053; 0.021

*The AF was taken as the reference group. ILs that were significant at the univariable analysis were further considered in the multivariable regression and logarithmic transformation was employed on these ILs to achieve symmetry in their respective distributions*.

* *Significant results with P < 0.05*.

Our results showed that when adjusting for age, gender, ever smoked, HDL, LDL, total Cholesterol, SBP and DBP, a significant difference in IL-4 level was detected between AF group and AF+CMS, and between AF and CMS group, the AF+CMS had reduced levels of IL4 by 2.19 units compared to AF group (*P* = 0.022) as shown in Table 4. Moreover, the CMS group also exhibited a decrease in IL-4 by 9.97 pg/mL compared to AF (*P* = 0.001). The clinical and demographic characteristics were not significantly associated with IL-4. With respect to IL-5 the adjusted analysis showed that IL-5 exhibited a significant difference between AF and CMS groups. The CMS group had a decrease in the mean IL-5 by 3.15 pg/mL (*P* = 0.031) compared to AF. Gender was shown to be significantly associated with IL-5 with females having a decrease in IL-5 by 1.99 units compared to males (*P* = 0.044). HDL was shown to have an incremental association with IL-5 whereby IL-5 increases by 3% when HDL increases by 1 pg/mL (*P* = 0.021). IL-10 was not significantly different between the different groups. Only HDL showed a significant incremental association with IL-10 whereby IL-10 increased by 1% when HDL increased by 1 mg/dL (*P* = 0.038). Age had a borderline significant inverse association with IL-10 (*P* = 0.059) whereby IL-10 was shown to decrease by about 1% when age increased by 1 year. IL-17F was shown to be significantly different between the AF and CMS group (*P* = 0.027). CMS exhibited a decrease in the mean IL-17F by 5.98 pg/mL compared to AF. SBP, HDL and LDL were also significantly associated with IL-17F (P-values were, respectively 0.047, 0.003, and 0.048) as shown in Table 3.

## Discussion

Recently, many studies emphasize a pathogenic link of inflammation to the development of AF and CMS. Here, we show that activated immune mediators play a key role in the pathogenesis of AF that is aggravated in combination with CMS due to an imbalance in pro-inflammatory profile and anti-inflammatory cytokine response. Th-17 cells, a unique subset of CD4+ T cells, are associated with an increase in IL-17A, IL-17F, IL-21, IL-22 and IFNγ production, and that these cytokines play important roles inflammation, autoimmunity, host defense, and tissue repair (Figure 1). IL-21 is a pleiotropic cytokine with effects on innate and adaptive immune cells and has been shown to promote Th-17 and Th-1 cells and inhibit Treg cells and has synergistic effects with IL-17A or IL-17F. IL-17A a pleiotropic pro-inflammatory cytokine, has been implicated in promoting a pro-inflammatory response and fibrosis and among signature Th-17 derived effector cytokines. Pro-inflammatory cytokines produced by immune cells such as IL-6 not only induce platelet activation but are also associated with adverse outcomes in AF patients. CRP has been considered a downstream marker of the inflammatory cascade, specific cytokines such as IL-6 and TNF-α have also been linked to AF. IL-6 is a pleotropic cytokine that mediates a variety of biological activities including pro-inflammatory responses and stimulates the Janus kinase/signal transducers and activators of transcription (JAK/STAT) pathway. IL-6 is produced by immune cells such as leukocytes and fibroblasts as well as non-immune cells such as endothelial cells, vascular smooth muscle cells and ischemic cardiomyocytes. Previous studies are consistent with our results showing an increase in IL-6 plasma levels in AF. TNF-α secreted by macrophages including those in fat and leukocytes induces activation of the transcription factor nuclear factor (NF)-kB. Thus, both cytokines exhibit a pleiotropic pro-inflammatory response, including myocyte and fibroblast differentiation, proliferation, and migration. Among other “adipokines,” IL-6 and TNF-α are also shown to promote CMS, thus, dysregulation of adipokine synthesis and release plays a critical role in insulin resistance. On the other hand, IL-4 is known to suppress the production of some inflammatory cytokines from immune cells and has been shown to promote tissue repair. The elevated levels of IL-4 in blood samples from AF patients compared to combined CMS AF patients highlights IL-4 role in anti-inflammatory responses; thus, less contribution to the inflammation in CMS AF patients. The balance between anti-inflammatory and inflammatory cytokines, such as IL-10 and TNF-α (tumor necrosis factor α), is also associated with AF recurrence. Inducing inhibitory markers or activating pathways that suppress inflammation including IL-10 may be protective and a potential therapeutic strategy for AF that could attenuate adverse cardiac effects.

Despite that both the electrophysiology and structural properties of the atria are critically affected by inflammatory processes, up till today, the anti-inflammatory drugs remain unsatisfactory. Various inflammatory cascades underlying AF may also differ between patients due to genetic polymorphisms such as IL-1, IL-6, and IL-10. For instance, AF patients with high levels of IL-6 owing to its gene polymorphism altering its gene expressions levels as we have shown int his study, did contributes to the pathogenesis of AF. Thus, novel therapies targeting IL-6 in addition to modification of other factors may prove to be beneficial for this disease.

In parallel, an accumulating body of evidence indicates that inflammatory pathways not only interfere with ion channel function of myocytes, but also regulate extracellular homogeneity of atrial tissue and fibrosis. Inflammation is a crucial indicator of fibrosis because of inflammatory signals such as NADPH oxidase, ROS production, cytokines, growth factors, angiotensin II as well as mechanical stretch provoke fibroblast proliferation, migration and differentiation into myofibroblasts. The latter are the principle subtypes in the diseased atrial myocardium to produce cytokines, TGF and MMPs. In this study, genotyping showed high percentages of patients with high secretor genotypes of IL-6 and TGF-β1.

The platelet-to-lymphocyte ratio (PLR) is considered as a new biomarker for predicting inflammation (18). Elevated platelets trigger the infiltration of neutrophils, monocytes, and lymphocytes to the vasculature and hence it is correlated with bad prognosis in CVD (19, 20). On the other hand, monocyte to high-density lipoprotein (HDL) ratio (MHR) is also an inflammatory biomarker predictive for many CVD (21). Monocyte-to-high density lipoprotein ratio (MHR) has been proposed as a novel prognostic indicator of cardiovascular diseases based on the pro-inflammatory effect of monocyte and anti-inflammatory effect of HDL. It has been reported to be related to cardiovascular outcomes in patients with chronic kidney disease and the recurrence of atrial fibrillation. In our present study, interestingly CMS in addition to AF (CMS +AF) displayed a higher MHR ratio suggesting a higher inflammatory response compared to CMS and AF alone. Red blood cell distribution width (RDW) measures the volume range of variation of red blood cells (RBC) (22). The pro-inflammatory state is associated with high RDW (23). As a consequence, RDW was found to be a significant predictor for the development of atrial fibrillation (24).

## Conclusion

A combination of both CMS and AF may be associated with a higher degree of inflammation in patients than what is seen in either CMS or AF alone. The discovery of a single inflammatory marker or inflammatory pathway contributing to AF may be a promising in the management of these diseases.

## Data Availability Statement

The original contributions presented in the study are included in the article/Supplementary Materials, further inquiries can be directed to the corresponding author/s.

## Ethics Statement

The studies involving human participants were reviewed and approved by AUB Institution Review Board. The patients/participants provided their written informed consent to participate in this study.

## Author Contributions

BA-S, MK, STA, SA, and MR helped in the patients' recruitment for this study. JE, MS, PZ, JB, AB, RI, MA, and MG helped in the human samples and data collection. HI, MA, AJ, and MR analyzed serum inflammatory cytokine and connective tissue growth factor levels. RM supervised the gene polymorphisms analysis of the inflammatory markers. MR, MAJ, and HI conceptualized the manuscript and the different sections included. MAJ carried out the statistical analysis of the data including the descriptive, non-parametric and multivariable analyses, prepared the tables that displayed the results of these analyses and the boxplots figures, and wrote and interpreted the statistical methods and results in the manuscript. MR supervised the study.

## Conflict of Interest

The authors declare that the research was conducted in the absence of any commercial or financial relationships that could be construed as a potential conflict of interest.
