# Supplementary material for: Genomic and Proteomic Study of the Inflammatory Pathway in Patients With Atrial Fibrillation and Cardiometabolic Syndrome
Source: Front Cardiovasc Med. 2020 Dec 2;7:613271. doi: 10.3389/fcvm.2020.613271 (PMC7738559; doi:10.3389/fcvm.2020.613271)
Supplement: Supplementary file 1 [file Table_1.docx]

Genomic and Proteomic Study of the Inflammatory Pathway in Patients with Atrial Fibrillation and Cardiometabolic Syndrome

Hana A. Itani ^1,2^, Miran A. Jaffa^3*^, Joseph Elias^4^, Mohammad Sabra^4^, Patrick Zakka^5^, Jad Ballout^4^, Amira Bekdash^1^, Rand Ibrahim^4^, Moustafa Al Hariri^6^, Mirna Ghemrawi^7^, Bernard Abi-Saleh^4^, Maurice Khoury^4^, Samir Alam^4^, Rami Mahfouz^7^, Ayad A. Jaffa^8^, Sami T Azar^9^, Marwan M. Refaat^4,8*^

**Supplementary Material**

**Appendix 1:** Summary statistics for the ILs (Table a), and Non-parametric Tests Results for IL Data (Kruskal Wallis and Mann Whitney U Tests Table b)

**Table a:** Descriptive statistics for the different interleukins (ILs) across the AF, AF+CMS, and CMS groups.

| **Descriptive Statistics:** | | | | | | |
| --- | --- | --- | --- | --- | --- | --- |
|  | **N** | **Mean** | **Median** | **Minimum** | **Maximum** | **Standard Deviation (SD)** |
| **IFN:** | | | | | | |
| AF | 18 | 4.032 | 3.009 | 1.075 | 13.155 | 3.073 |
| AF+CMS | 30 | 6.392 | 3.045 | 1.345 | 13.353 | 7.786 |
| CMS | 10 | 3.152 | 3.026 | 2.159 | 4.416 | 0.969 |
| **TNF:** | | | | | | |
| AF | 17 | 3.074 | 2.835 | 1.072 | 8.364 | 1.880 |
| AF+CMS | 29 | 4.1813 | 3.945 | 0.351 | 13.198 | 3.594 |
| CMS | 11 | 3.405 | 1.937 | 0.982 | 7.681 | 2.634 |
| **IL_2:** | | | | | | |
| AF | 14 | 0.463 | 0.256 | 0.025 | 1.809 | 0.544 |
| AF+CMS | 22 | 0.404 | 0.144 | 0.006 | 2.181 | 0.587 |
| CMS | 10 | 0.385 | 0.112 | 0.007 | 1.901 | 0.584 |
| **IL_4:** | | | | | | |
| AF | 23 | 4.290 | 3.802 | 0.410 | 11.685 | 2.884 |
| AF+CMS | 31 | 2.100 | 1.017 | 0.344 | 7.379 | 1.859 |
| CMS | 10 | 0.874 | 0.665 | 0.507 | 1.929 | 0.465 |
| **IL_5:** | | | | | | |
| AF | 25 | 4.497 | 4.436 | 1.175 | 10.249 | 2.509 |
| AF+CMS | 30 | 4.750 | 3.186 | 0.357 | 15.176 | 4.098 |
| CMS | 10 | 2.033 | 1.498 | 1.072 | 3.999 | 0.968 |
| **IL_6:** | | | | | | |
| AF | 20 | 5.166 | 4.515 | 1.366 | 10.726 | 2.751 |
| AF+CMS | 29 | 8.656 | 4.825 | 0.563 | 35.264 | 9.107 |
| CMS | 10 | 4.889 | 4.576 | 1.107 | 14.334 | 3.764 |
| **IL_9:** | | | | | | |
| AF | 20 | 2.426 | 1.771 | 0.215 | 7.590 | 1.910 |
| AF+CMS | 30 | 3.166 | 2.123 | 0.390 | 12.051 | 3.017 |
| CMS | 10 | 1.574 | 1.311 | 0.504 | 3.398 | 0.848 |
| **IL_10** | | | | | | |
| AF | 24 | 2.167 | 2.070 | 0.921 | 3.547 | 0.815 |
| AF+CMS | 31 | 1.833 | 1.493 | 0.744 | 5.110 | 1.117 |
| CMS | 10 | 1.166 | 1.176 | 0.960 | 1.358 | 0.150 |
| **IL_13:** | | | | | | |
| AF | 16 | 14.367 | 8.284 | 0.299 | 37.731 | 13.899 |
| AF+CMS | 7 | 4.718 | 5.397 | 0.006 | 11.532 | 4.438 |
| CMS | 1 | - | - | - | - | - |
| **IL_17A:** | | | | | | |
| AF | 15 | 0.579 | 0.295 | 0.022 | 2.684 | 0.824 |
| AF+CMS | 17 | 0.785 | 0.272 | 0.005 | 5.903 | 1.466 |
| CMS | 4 | 0.089 | 0.083 | 0.009 | 0.182 | 0.076 |
| **IL_17F:** | | | | | | |
| AF | 22 | 2.052 | 1.596 | 0.062 | 7.537 | 1.935 |
| AF+CMS | 30 | 1.370 | 0.556 | 0.098 | 6.563 | 1.802 |
| CMS | 11 | 0.332 | 0.351 | 0.120 | 0.770 | 0.196 |
| **IL_21:** | | | | | | |
| AF | 9 | 12.616 | 11.870 | 1.649 | 24.442 | 8.531 |
| AF+CMS | 20 | 9.165 | 4.575 | 0.011 | 29.110 | 8.869 |
| CMS | 8 | 5.304 | 5.217 | 0.463 | 13.732 | 4.477 |
| **IL_22:** | | | | | | |
| AF | 24 | 4.028 | 2.671 | 0.018 | 15.441 | 4.508 |
| AF+CMS | 25 | 3.662 | 1.532 | 0.247 | 15.948 | 4.639 |
| CMS | 10 | 1.078 | 0.465 | 0.035 | 4.826 | 1.475 |

**Table b:** Results of the statistical analysis using non-parametric tests to compare the interleukin values between all the three groups (AF, AF+CMS, CMS).

| Statistical Test: | Interleukin | Compared Groups | | |
| --- | --- | --- | --- | --- |
|  |  | **AF and AF+CMS (P-values)** | **AF and CMS (P-values)** | **AF+MS and CMS (P-values)** |
| Mann- Whitney U Test P-value Unadjusted | IFN | 0.717 | 0.701 | 0.617 |
|  | TNF | 0.133 | 0.707 | 0.250 |
|  | IL_2 | 0.506 | 0.539 | 0.839 |
|  | IL_4 | 0.001* | 0.000* | 0.060 |
|  | IL_5 | 0.566 | 0.004* | 0.070 |
|  | IL_6 | 0.483 | 0.582 | 0.357 |
|  | IL_9 | 0.488 | 0.312 | 0.115 |
|  | IL_10 | 0.035* | 0.002* | 0.039* |
|  | IL_13 | 0.095 | --- | --- |
|  | IL_17A | 0.533 | 0.080 | 0.303 |
|  | IL_17F | 0.039* | 0.001* | 0.130 |
|  | IL_21 | 0.258 | 0.068 | 0.476 |
|  | IL_22 | 0.992 | 0.076 | 0.021 |
| Mann-Whitney U Test P-value Adjusted for Multiple Comparisons | IL_4 | 0.006* | 0.000* | 0.256 |
|  | IL_5 | 1.000 | 0.034* | 0.110 |
|  | IL_10 | 0.160 | 0.003* | 0.160 |
|  | IL_17F | 0.102 | 0.005* | 0.326 |
| Kruskal Wallis P-value | IFN | 0.841 | | |
|  | TNF | 0.250 | | |
|  | IL_2 | 0.750 | | |
|  | IL_4* | 0.000* | | |
|  | IL_5* | 0.037* | | |
|  | IL_6 | 0.566 | | |
|  | IL_9 | 0.273 | | |
|  | IL_10* | 0.003* | | |
|  | IL_13 | Kruskal Wallis test was not used since MS group was dropped out of the analysis as it had only 1 observation | | |
|  | IL_17A | 0.272 | | |
|  | IL_17F* | 0.005* | | |
|  | IL_21 | 0.228 | | |
|  | IL_22 | 0.084 | | |

* Statistical Significance was reached

**Appendix 2:** Box Plots and explanation of the distributions of the ILs that were significantly different across the groups (AF, AF+MS, MS) at the crude non-parametric analysis.

**Figure 1 (a-e):** Box Plot for IL-4 (a), IL-5(b), IL-10(c), IL-17F(d), and IL-22 (e) values in the AF, AF+CMS, and CMS groups. The Box plots of the IL values corresponding to the three groups (AF, AF+CMS, and MS) showed presence of outliers and skewed distribution of ILs. Accordingly, given the relatively small sample size in each group and the non-normal distribution of the ILs in the groups, we resorted to applying the non-parametric tests to determine if there was a significant difference in IL values between the three different groups, and therefore identifying if there is any group effect.
